# A useful and safe method for retrieving a round metallic object from an airway

**DOI:** 10.1002/ccr3.3634

**Published:** 2020-12-10

**Authors:** Daisuke Jingu, Akira Horii, Yu Matsuzono, Satoshi Ubukata, Kosuke Sato, Makoto Shoji, Noriko Kondo, Hiroshi Takahashi, Hiroshi Watanabe

**Affiliations:** ^1^ Department of Respiratory Medicine Saka General Hospital Shiogama Japan; ^2^ Department of Internal Medicine Saka General Hospital Shiogama Japan; ^3^ Department of Anesthesiology Saka General Hospital Shiogama Japan

**Keywords:** airway foreign body, laryngeal mask, metallic round body, net forceps

## Abstract

The endoscopic net forceps with the support of a laryngeal mask airway are a dependable choice for retrieving a round metallic object from an airway.

## Graphical abstract

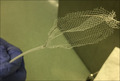

## 1 INTRODUCTION

We experienced a case with accidental aspiration of a metallic round foreign body. As there is no established technique for retrieving a metallic round ball from an airway, we propose that endoscopic net forceps with the support of a laryngeal mask airway are the best choice for such occasions.

## 2 CLINICAL IMAGE

**Figure 1:**
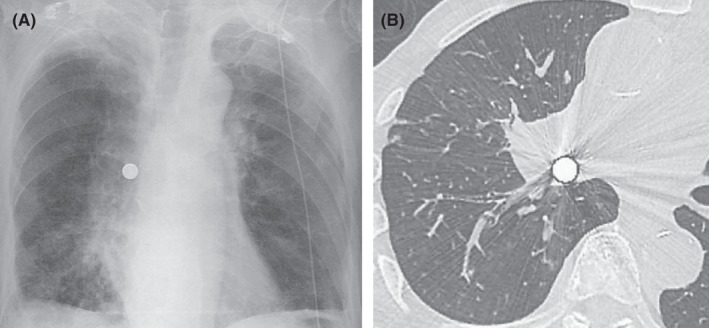
Chest radiograph (A) and CT scan (B) show a round high‐density shadow nearly 1cm in diameter. The right inferior lobar bronchus seems to be obstructed by a metallic round substance.

**Figure 2:**
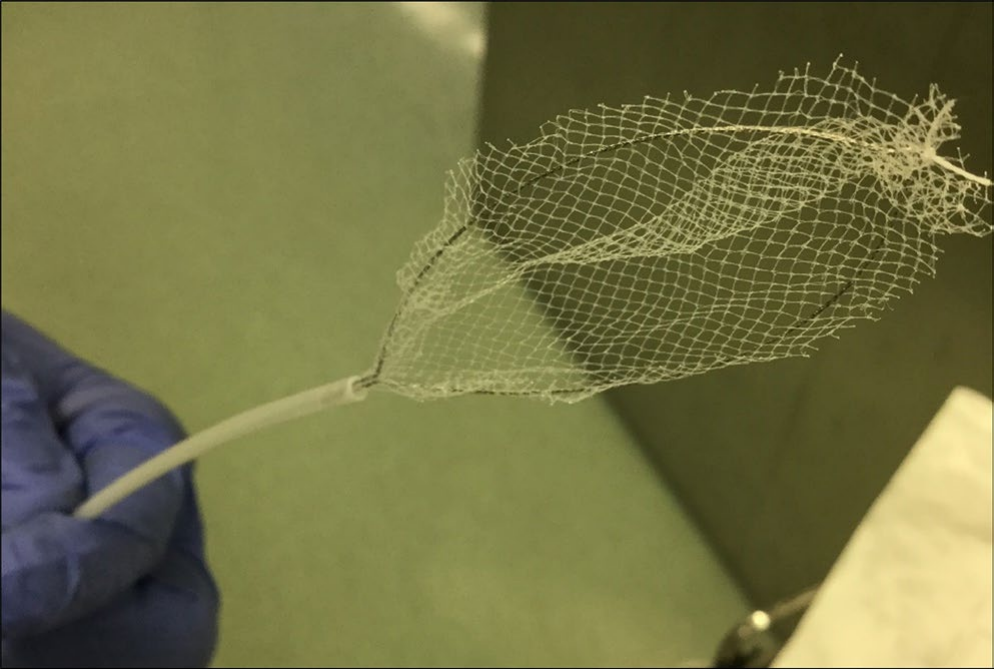
The net forceps we used are shown (Roth Net^®^—foreign body—standard, US Endoscopy, Mentor, OH). This picture shows the net forceps at the open status. The foreign body can be trapped inside of the net (also see Figure 3B).

**Figure 3:**
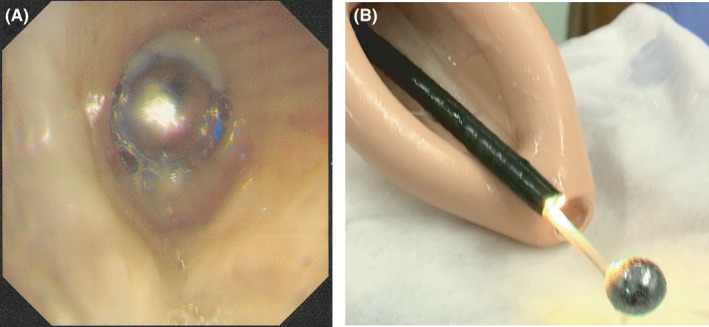
(A) Bronchoscopic examination revealed complete obstruction of the right inferior lobar bronchus by a metallic round foreign body. (B) The foreign body was successfully removed by endoscopic net forceps with the support of a laryngeal mask airway. The foreign body was a pachinko ball.

An 85‐year‐old man with a 2‐day history of dyspnea and general malaise was admitted to our hospital with hypopnea and acidosis. Chest radiograph and CT scan identified a round high‐density shadow about 1 cm in diameter (Figure 1). We deduced that a pachinko ball (Pachinko is a Japanese pinball gambling game) obstructed the right inferior lobar bronchus. Forced coughing and back blow failed to remove it. To remove the obstacle with minimal invasion, we considered several devices with an endoscope under mechanical ventilation: a magnet, a basket type or a pentatope type grasping forceps, or an endoscopic net forceps (Figure 2), with the support of a laryngeal mask airway, which we selected. The pachinko ball was successfully retrieved without severe damage to the patient (Figure 3). The patient received noninvasive positive‐pressure ventilation for one day and antibiotic administration for 14 days. Rehabilitation improved his general condition, and he was transferred to a long‐term hospital on day 48. There is no consensus on how to retrieve a metallic round ball from an airway. Based on our experience, we propose that endoscopic net forceps with the support of a laryngeal mask airway are a dependable choice for such occasions.

## CONFLICT OF INTEREST

None declared.

## **AUTHOR** **CONTRIBUTIONS**

DJ, YM, SU, KS, MS, HT, HW, and NK: substantially contributed to the diagnosis and clinical care of the patient; DJ: wrote the draft of the manuscript; DJ and AH: critically revised it for important intellectual content; HW: gave final approval of the version to be published.

## Data Availability

No data available in this case report.

